# Cannabis use as a potential mediator between childhood adversity and first-episode psychosis: results from the EU-GEI case–control study

**DOI:** 10.1017/S0033291723000995

**Published:** 2023-11

**Authors:** Giulia Trotta, Victoria Rodriguez, Diego Quattrone, Edoardo Spinazzola, Giada Tripoli, Charlotte Gayer-Anderson, Tom P Freeman, Hannah E Jongsma, Lucia Sideli, Monica Aas, Simona A Stilo, Caterina La Cascia, Laura Ferraro, Daniele La Barbera, Antonio Lasalvia, Sarah Tosato, Ilaria Tarricone, Giuseppe D'Andrea, Andrea Tortelli, Franck Schürhoff, Andrei Szöke, Baptiste Pignon, Jean-Paul Selten, Eva Velthorst, Lieuwe de Haan, Pierre-Michel Llorca, Paulo Rossi Menezes, Cristina M Del Ben, Jose Luis Santos, Manuel Arrojo, Julio Bobes, Julio Sanjuán, Miquel Bernardo, Celso Arango, James B Kirkbride, Peter B Jones, Alexander Richards, Bart P Rutten, Jim Van Os, Isabelle Austin-Zimmerman, Zhikun Li, Craig Morgan, Pak C Sham, Evangelos Vassos, Chloe Wong, Richard Bentall, Helen L Fisher, Robin M Murray, Luis Alameda, Marta Di Forti, EU-GEI WP2 Group

**Affiliations:** 1 Social, Genetic and Developmental Psychiatry Centre, Institute of Psychiatry, Psychology and Neuroscience, King's College London, London, UK; 2 Department of Psychosis Studies, Institute of Psychiatry, Psychology and Neuroscience, King's College London, London, UK; 3 Health Service and Population Research, Institute of Psychiatry, Psychology, and Neuroscience, King's College London, London, UK; 4 University of Bath Department of Pharmacy and Pharmacology: University of Bath Department of Life Sciences, Bath, UK; 5 PsyLife Group, Division of Psychiatry, University College London, London, UK; 6 Department of Human Science, LUMSA University, Rome, Italy; 7 University of Palermo Department of Biomedicine Neuroscience and Advanced Diagnostics: Universita degli Studi di Palermo Dipartimento di Biomedicina Neuroscienze e Diagnostica avanzata, Palermo, Italy; 8 Section of Psychiatry, Department of Neuroscience, Biomedicine and Movement, University of Verona, Verona, Italy; 9 University of Bologna Department of Medical and Surgical Sciences: Universita degli Studi di Bologna Dipartimento di Scienze Mediche e Chirurgiche, Bologna, Italy; 10 Establissement Public de Sante, Maison Blanche, France; 11 Univ Paris Est Creteil (UPEC), AP-HP, Hopitaux Universitaires ‘H. Mondor’, DMU IMPACT, INSERM, IMRB, Translational Neuropsychiatry, Fondation FondaMental, F-94010 Creteil, France; 12 Institute for Mental Health, GGZ Rivierduinen, Leiden, The Netherlands; 13 Mount Sinai School of Medicine Department of Psychiatry: Icahn School of Medicine, New York, NY, USA; 14 Early Psychosis Section, Department of Psychiatry, Amsterdam UMC, Amsterdam, The Netherlands; 15 EA 7280 Npsydo, Universite Clermont Auvergne, Clermont-Ferrand, France; 16 Department of Preventive Medicine, Faculdade de Medicina, Universidade de São Paulo, São Paulo, Brazil; 17 Department of Neuroscience and Behaviour, Division of Psychiatry, Ribeirao Preto Medical School, University of São Paulo, São Paulo, Brazil; 18 Department of Psychiatry, Hospital ‘Virgen de la Luz’, Cuenca, Spain; 19 Department of Psychiatry, Psychiatric Genetic Group, Instituto de Investigation Sanitaria de Santiago de Compostela, Complejo Hospitalario Universitario de Santiago de Compostela, Spain; 20 Department of Medicine, Psychiatry Area, Universidad de Oviedo, ISPA, INEUROPA, CIBERSAM, Oviedo, Spain; 21 Department of Psychiatry, Centro de Investigation Biomedica en Red de Salud Mental, School of Medicine, Universidad de Valencia, Spain; 22 Barcelona Clinic Schizophrenia Unit, Hospital Clinic, Department of Medicine, Neuroscience Institute, University of Barcelona, Institute d'investigations Biomediques, August Pi I Sunyer, Centro de Investigation Biomedica en Red de Salud Mental, Barcelona, Spain; 23 Department of Child and Adolescent Psychiatry, Institute of Psychiatry and Mental Health, Hospital General Universitario Gregorio Maranon, School of Medicine, Universidad Complutense, ISGM, CIBERSAM, Madrid, Spain; 24 CAMEO Early Intervention Service, Cambridgeshire and Peterborough National Health Service Foundation Trust, Cambridge, England; 25 Division of Psychological Medicine and Clinical Neurosciences, MRC Centre for Neuropsychiatric Genetics and Genomics, Cardiff University, Cardiff, UK; 26 Department of Psychiatry and Neuropsychology, School for Mental Health and Neuroscience, Maastricht University, Maastricht, The Netherlands; 27 Hong Kong University: University of Hong Kong, Hong Kong; 28 The University of Sheffield Department of Psychology, Sheffield, UK

**Keywords:** Cannabis use, childhood experience, mediation, psychotic disorders, trauma

## Abstract

**Background:**

Childhood adversity and cannabis use are considered independent risk factors for psychosis, but whether different patterns of cannabis use may be acting as mediator between adversity and psychotic disorders has not yet been explored. The aim of this study is to examine whether cannabis use mediates the relationship between childhood adversity and psychosis.

**Methods:**

Data were utilised on 881 first-episode psychosis patients and 1231 controls from the European network of national schizophrenia networks studying Gene–Environment Interactions (EU-GEI) study. Detailed history of cannabis use was collected with the Cannabis Experience Questionnaire. The Childhood Experience of Care and Abuse Questionnaire was used to assess exposure to household discord, sexual, physical or emotional abuse and bullying in two periods: early (0–11 years), and late (12–17 years). A path decomposition method was used to analyse whether the association between childhood adversity and psychosis was mediated by (1) lifetime cannabis use, (2) cannabis potency and (3) frequency of use.

**Results:**

The association between household discord and psychosis was partially mediated by lifetime use of cannabis (indirect effect coef. 0.078, s.e. 0.022, 17%), its potency (indirect effect coef. 0.059, s.e. 0.018, 14%) and by frequency (indirect effect coef. 0.117, s.e. 0.038, 29%). Similar findings were obtained when analyses were restricted to early exposure to household discord.

**Conclusions:**

Harmful patterns of cannabis use mediated the association between specific childhood adversities, like household discord, with later psychosis. Children exposed to particularly challenging environments in their household could benefit from psychosocial interventions aimed at preventing cannabis misuse.

## Introduction

A growing body of literature has investigated the nature of the association between childhood adversity and psychosis (Belbasis et al., 2018; Morgan & Gayer-Anderson, 2016). In recent years multiple possible mediating mechanisms have been suggested in the adversity–psychosis association, including the role of mood, PTSD-related symptoms, negative schemas about the self, the world and others (Alameda et al., 2020). Identifying possible treatable factors that contribute to this association may have important clinical implications, as they can constitute the object of specific interventions that may decrease the negative impact of adversity in those with a psychotic disorder (Bebbington, 2015).

Both childhood adversity and cannabis use increased the risk of psychosis independently [OR 2.8 for the former (Varese et al., 2012) and 3.9 for the latter (Marconi, Di Forti, Lewis, Murray, & Vassos, 2016)]. There is also evidence showing that the coexistence between both risk factors have an interactive effect on the risk of the disorder (Bentall, Wickham, Shevlin, & Varese, 2012; Harley et al., 2010; Houston, Murphy, Adamson, Stringer, & Shevlin, 2008; Konings et al., 2012; Sideli et al., 2018). Moreover, some reports have suggested that cannabis use may be a consequence of childhood adversity exposure (Morgan et al., 2014; Van Nierop et al., 2014). This leads to the possible scenario where some individuals at risk for the disorder who have been exposed to childhood adversity may use cannabis as a maladaptive coping strategy to cope with the unpleasant emotions led by trauma. In this line, some studies have examined whether cannabis may be a mediator between adversity and psychotic symptoms (Etain et al., 2017; Frydecka et al., 2020; Van Nierop et al., 2014). An online survey conducted in Poland investigated the relationship between early trauma, cognitive biases, cannabis use and risk of psychosis among young adults from the general population (Frydecka et al., 2020). The results showed an important mediating role of cannabis use and cognitive biases in the association between childhood traumatic events and the development of psychotic-like experiences. Van Nierop et al. aimed to elucidate the effect of social defeat, cannabis use and affective dysregulation on the association between childhood trauma and psychosis in a large population-based sample (van Nierop et al., 2014). While both social defeat and affective dysregulation acted as separate mediators, cannabis use did not mediate this association. Another study found no evidence of mediation between various childhood adversities and bipolar disorder, via cannabis use (Etain et al., 2017).

To the best of our knowledge, there are no studies testing this hypothesis in patients with a psychotic disorder, and none exploring detailed measures of cannabis use such as the potency and frequency of use, and taking into account the varying influence of different forms of childhood adversity. Given that cannabis use can potentially be modified by prevention or treatment (Lees et al., 2021), more large epidemiological studies testing its possible mediating contribution between adversity and psychosis are of great interest, as preventing or reducing cannabis use could potentially reduce the harmful effect of childhood adversity on psychosis.

Based on the above, using data from the European network of national schizophrenia networks studying Gene–Environment Interactions (EU-GEI) case–control study of first-episode psychosis, we tested whether the relationship between childhood adversity and psychotic disorder is mediated by (i) lifetime cannabis use, (ii) cannabis potency and (iii) frequency of using cannabis. We hypothesised that cannabis use indirectly underlined the link between different types of childhood adversity and psychosis. If our hypothesis is confirmed, it would help clinicians to identify ‘at-risk’ individuals that could be targeted for specific preventive and therapeutic interventions.

## Methods

The ‘first-episode’ work package of the EU-GEI study consists of a multicentre incidence and case–control study of genetic and environmental determinants of psychotic disorders (Jongsma et al., 2018), including 17 catchment areas across six countries: England (*n* = 2; southeast London, Cambridgeshire and Peterborough), France (*n* = 3; 20th arrondissement of Paris, Val-de-Marne, Puy-de-Dôme), the Netherlands (*n* = 2; central Amsterdam, Gouda and Voorhout), Italy (*n* = 3; part of the Veneto region, Bologna municipality and the city of Palermo), Spain (*n* = 6; Madrid, Barcelona, Valencia, Oviedo, Santiago and Cuenca) and Brazil (*n* = 1; Ribeirão Preto).

### Participants

All individuals who contacted mental health services in the 17 catchment areas over a median case ascertainment period of 25 months (interquartile range: 24–36 months) (Jongsma et al., 2018), between 1 May 2010 and 1 April 2015, with a suspected first episode of psychosis (FEP) were identified by trained researchers, and included if they met the following criteria: (i) being a resident within the catchment area at first presentation; (ii) aged 18–64 years and (iii) presented to services with a clinical diagnosis of psychosis (*International Statistical Classification of Diseases, Tenth Revision* [*ICD-10*] codes F20–33) (World Health Organization, 1992). Patients were excluded if they had previous contact with mental health services for psychosis, evidence of psychotic symptoms precipitated by an organic cause or resulting from acute intoxication, as defined by the ICD-10 (codes F1x.5). Controls were recruited from the general population living in the same catchment areas using a quota sampling approach to maximise representativeness by age, sex and ethnicity (Gayer-Anderson et al., 2020). Controls were excluded if they had received a diagnosis of, or treatment for, psychotic disorder. The authors assert that all procedures contributing to this work comply with the ethical standards of the relevant national and institutional committees on human experimentation and with the Helsinki Declaration of 1975, as revised in 2008. All participants provided informed, written consent; and ethical approval was provided by research ethics committees in each site: South London and Maudsley and Institute of Psychiatry Research Ethics Committee; National Research Ethics Service Committee East of England–East Cambridge; Medisch-Ethische Toetsingscommissie van het Academisch Centrum te Amsterdam; Comité Ético de Investigación Clínica Hospital Gregorio Marañón; Comité Ético de Investigación Clínica del Hospital Clinic de Barcelona; Comité Ético de Investigación Clínica del Hospital Clinic Universitari de Valencia; Comité Ética de la Investigación Clínica del Principado de Asturias; Comité Ético de Investigación Clínica de Galicia; Comité Ético de Investigación Clínica del Hospital Virgen de la Luz de Cuenca; Comité de Protéction des Personnes–CPP Île de France IX; Comitato Etico Policlinico S Orsola Malpighi; Comitato Etico Azienda Ospedaleria Universitaria di Verona; Comitato Etico Palermo 1, Azienda Ospedaliera Policlinico ‘Paolo Giaccone’ and Research Ethics Committee of the clinical Hospital of Ribeirão Preto Medical School, University of São Paulo, Brazil.

Given that our aim is to explore the relation between early childhood adversity and later cannabis use, in order to reduce the influence of reverse causality, we excluded subjects that had used cannabis before the age of 12 years old.

### Measures

#### Sociodemographic data

We obtained sociodemographic data during interviews with participants using the Medical Research Council Sociodemographic Schedule (Mallett, 1997). In this study, we used age, sex, ethnicity (white, black, Mixed, Asian, north African and other), country and years of education.

#### Childhood adversity

The Childhood Experience of Care and Abuse (CECA) and a specific questionnaire on bullying were read out to participants during a face-to-face interview. The CECA is an instrument developed to retrospectively assess childhood adversity that occurred before 18 years of age. In this study, we focused on experiences of childhood abuse (sexual, physical and psychological) and household discord reported as occurring between ages 0 and 17 years (Roy & Perry, 2004). Psychological abuse comprised humiliation, degradation, extreme rejection, emotional blackmail, terrorizing by a caregiver or deprivation of basic needs. Physical abuse was defined as bodily harm inflicted by a caregiver that resulted in at least bruising. Sexual abuse was defined as the participant's report of any unwanted sexual incident. Household discord was defined as the amount of fighting between the caregivers and/or with the child. For bullying, the participants were asked if they had experienced any of the following from peers before 17 years of age: having been verbally abused or made fun of; having been ignored, excluded or left out on purpose; having been hit, kicked, shoved or locked in a room; having been told lies or been the subject of false rumours; having been a victim of any other type of bullying. For the analyses, each childhood adversity subtype was dichotomised based on severity cut-off thresholds: ‘absent’ – (0) if none or some – or ‘present’ – (1) if moderate or marked.

Sensitivity analyses were conducted using age at the time of the first exposure which was categorised as follows: (1) early adversity refers to exposure between birth and age 11 years, (2) late adversity refers to exposure between ages 12 and 17 years, as has been done previously (Alameda et al., 2017).

#### Cannabis use

We utilised an updated version of the modified Cannabis Experience Questionnaire (CEQ_EU−GEI_) to collect a detailed history of cannabis use from participants (Di Forti et al., 2009). Following from the EUGEI study cannabis core paper (Di Forti et al., 2019), we included three measures of cannabis use in the analyses: (i) lifetime cannabis use (which in this paper includes those starting cannabis from age 12), (ii) lifetime frequency of use and (iii) cannabis potency. Among cannabis users, we selected subjects who started using cannabis in adolescence (i.e. after age 12) in order to clarify the temporal relationship among childhood adversity and later exposure to cannabis.

Lifetime frequency of use was categorised as (1) used never or occasionally, (2) used more than once a week and (3) daily. The cannabis potency was dichotomised based on the expected amount of THC that subjects reported to have used: (1) low potency cannabis with less than 10% of THC, and (2) high potency more than 10% of THC (see online Supplementary Methods).

#### Diagnostic assessment

We obtained research-based diagnoses based on the Operational Criteria Checklist algorithm (OPCRIT), with good inter-rater reliability across catchment areas (*κ* = 0.7). The OPCRIT system allows researchers to: (i) assess the pre-morbid history and current mental state; and (ii) establish the diagnosis of psychotic disorders based on algorithms for several diagnostic classification systems. It consists of a 96-item checklist which can be filled based on a semi-structured clinical interview or review of clinical notes and other relevant information (McGuffin, Farmer, & Harvey, 1991). Where OPCRIT assessment was not possible, we relied on clinical diagnoses.

### Statistical analysis

Case–control comparisons on sociodemographics and primary outcomes measures (cannabis use and childhood adversity) were made with χ^2^, Student *t* or Wilcoxon–Mann–Whitney tests depending on normality of data distribution. We used mediation analyses to test whether the relationship between childhood adversities and psychosis is direct or whether putative mediator variables (i.e. lifetime cannabis use, cannabis potency and lifetime frequency of use) account for the relationship between them (see online Supplementary Fig. S1). Sensitivity analyses were conducted examining the mediational role of cannabis in the association between early/late exposure to different types of adversity and first-episode psychosis.

Following Baron and Kenny's (Baron & Kenny, 1986) criteria, running logistic regressions we first ascertained that: (i) there is an association between different subtypes of childhood adversity and psychotic disorder (pathway c); (ii) the putative mediator variables are associated with psychotic disorder (pathway b) and (iii) there is an association between childhood adversity and cannabis use (pathway a). We then performed mediation modelling using the Karlson, Holm and Breen method as framed in the *khb* package in Stata 15. Each putative mediator was entered in separate models to investigate their individual impact on the overall relationship. If the entry of the mediator was accompanied by a statistically significant effect on the dependent variable together with a reduction of the childhood adversity effect, mediation can be deduced. On the other hand, if the effect of the mediator lacked statistical significance, all that can be inferred is a direct effect of childhood adversity on psychosis. Analyses were controlled for age, sex, ethnicity, years of education, country and other childhood adversities. All statistical tests were two-tailed and significance was determined at the 0.05 level.

## Results

### Sample

From the original sample of 1130 cases and 1499 controls, we excluded 491 subjects (229 first-episode psychosis patients and 262 patients) for missing data on the variables of interest, and 26 participants (20 controls and six cases) for starting using cannabis before 12, leaving a final sample composed of 1231 controls and 881 cases (see online Supplementary results for more detailed recruitment flow-charts).

### Rates of childhood adversities, cannabis use and sociodemographic characteristics

Sociodemographic characteristics, rates of childhood adversities and cannabis use are described in Table 1.

**Table 1.** Sociodemographics, childhood adversities and cannabis use across all included first-episode psychosis cases and unaffected controls

	Total, *n* *=* 2112	Controls, *n* *=* 1231	Cases, *n* *=* 881	Controls *v*. cases
Age in years, *M* (s.d.)	33.97 (12.58)	36.19 (13.43)	30.88 (10.55)	*U* = 8.749*
Sex, male, *N* (%)	1124 (53.23)	578 (46.99)	546 (61.98)	χ*^2^* *=* 46.2906*
Ethnicity, *N* (%) White Black Mixed Asian North African Other	1447 (68.61) 277 (13.13) 215 (10.19) 64 (3.03) 65 (3.08) 41 (1.94)	927 (75.43) 115 (9.36) 112 (9.11) 33 (2.69) 23 (1.87) 19 (1.55)	520 (59.09) 162 (18.41) 103 (11.70) 31 (3.52) 42 (4.77) 22 (2.50)	χ*^2^* *=* 72.9084*
Years of education, *M* (s.d.)	15.24 (10.37)	15.32 (7.43)	15.12 (13.43)	*U* = 8.462*
Country, *N* (%) UK Holland Spain France Italy Brazil	571 (27.04) 402 (19.03) 143 (6.77) 214 (10.13) 292 (13.83) 490 (23.20)	334 (27.13) 210 (17.06) 75 (6.09) 147 (11.94) 165 (13.40) 300 (24.37)	237 (26.90) 192 (21.79) 68 (7.72) 67 (7.60) 127 (14.42) 190 (21.57)	χ*^2^* *=* 19.7118*
Childhood trauma, *N* (%) Household discord Psychological abuse Physical abuse Sexual abuse Bullying	932 (44.81) 259 (12.50) 556 (26.77) 206 (9.94) 732 (36.08)	481 (39.27) 111 (9.09) 291 (23.79) 102 (8.33) 371 (31.02)	451 (52.75) 148 (17.39) 265 (31.03) 104 (12.26) 361 (43.34)	χ*^2^* *=* 30.8554*
Childhood trauma, *N* (%) Early trauma Late trauma	851 (43.11) 511 (25.89)	488 (41.22) 273 (23.06)	363 (45.95) 238 (30.13)	χ*^2^* *=* 32.8989*
Lifetime cannabis use, yes *N* (%)	1133 (45.07)	568 (46.22)	565 (64.13)	χ*^2^* *=* 69.6654*
Age first tried cannabis, *M* (s.d.)	17.51 (4.58)	18.04 (4.60)	16.97 (4.50)	*U* = 5.416*
Cannabis potency, *N* (%) Non-users Low potency (THC <10%) High potency (THC ⩾10%)	951 (48.05) 631 (31.88) 397 (20.06)	648 (55.86) 341 (29.40) 171 (14.74)	303 (37.00) 290 (35.41) 226 (27.59)	χ*^2^* *=* 80.5330*
Cannabis frequency, *N* (%) Never or occasional More than once a week Daily	604 (53.88) 190 (16.95) 327 (29.17)	398 (70.19) 90 (15.87) 79 (13.93)	206 (37.18) 100 (18.05) 248 (44.77)	χ*^2^* *=* 148.7712*

Early trauma refers to exposure between birth and age 11; late trauma refers to exposure between ages 12 and 17; THC, Δ⁹-tetrahydrocannabinol; *U*, Mann–Whitney *U* test; χ*^2^*, Chi-squared test; *p value ⩽0.05.

As a whole, the mean age of cases was 30.88 (s.d. 10.55), 61.98% of which were male. There were differences in terms of sociodemographic characteristics between psychosis cases and controls. Patients were younger, more often men, with a lower level of education, and from ethnic minorities (all *p*'s <0.05). Among the 2112 participants, 1362 (69.00%) had been exposed to at least one of the selected adversities (breakdown of prevalence by each subtype is presented in Table 1); of these 851 (62.48% of exposed participants) had been exposed before age 12 (early adversity) and 511 (37.52% of exposed participants) between age 12 and 17 (late adversity). Patients reported a higher rate of childhood adversities considering all different subtypes, i.e. household discord, psychological abuse, physical abuse, sexual abuse and bullying (all *p*'s <0.05). A higher proportion of cases than controls significantly reported having ever used cannabis, 64.13% and 46.22%, respectively. We also found differences between cases and controls in terms of pattern of use (see Table 1) and age at first tried cannabis (see Fig. S4) (*p* < 0.05). Indeed, cases often reported a more harmful pattern of use (i.e. high potency and weekly/daily use) and started using cannabis earlier in adolescence.

### Associations between childhood adversity and cannabis use with psychotic disorder

Results of logistic regression analyses of both childhood adversity and cannabis use on psychosis are shown in Table 2 (panel A).

**Table 2.** Associations between childhood adversities and cannabis use with psychotic disorder (panel A) and associations between childhood adversity and cannabis use (panel B)

Panel A. Logistic regressions between childhood adversities, cannabis use and psychotic disorder		
	aOR (95% CI)	*p* value
Household discord Total adversity Early adversity	**1.53** (1.25–1.88)^a^ **1.67** (1.31–2.14)^a^	<0.001^a^ <0.001^a^
Psychological abuse Total adversity Early adversity	**1.79** (1.30–2.47)^a^ **1.59** (1.02–2.47)^a^	<0.001^a^ 0.039^a^
Physical abuse Total adversity Early adversity	0.86 (0.67–1.10)^a^ 0.84 (0.62–1.14)^a^	0.240^a^ 0.250^a^
Sexual abuse Total adversity Early adversity	**1.42** (1.01–2.00)^a^ 1.43 (0.90–2.27)^a^	0.042^a^ 0.133^a^
Bullying Total adversity Early adversity	**1.57** (1.27–1.96)^a^ **1.54** (1.17–2.02)^a^	<0.001^a^ 0.002^a^
Lifetime cannabis use	**2.04** (1.62–2.58)^b^	<0.001^b^
Cannabis potency	**1.53** (1.32–1.76)^b^	<0.001^b^
Cannabis frequency	**2.93** (2.24–3.84)^b^	<0.001^b^

Panel B. Logistic regressions between childhood adversities and cannabis use						
	Lifetime cannabis use		Cannabis potency		Cannabis frequency	
	aOR (95% CI)^a^	*p* value^a^	aOR (95% CI)^a^	*p* value^a^	aOR (95% CI)^a^	*p* value^a^
Household discord Total adversity Early adversity	**1.76** (1.37–2.25) **1.80** (1.33–2.44)	<0.001 <0.001	**1.47** (1.19–1.83) **1.33** (1.02–1.74)	<0.001 0.033	**1.71** (1.30–2.25) **1.79** (1.27–2.53)	<0.001 0.001
Psychological abuse Total adversity Early adversity	1.33 (0.89–1.98) **1.92** (1.10–3.38)	0.168 0.023	1.39 (0.98–1.97) **1.86** (1.14–3.04)	0.067 0.013	1.29 (0.85–1.96) 1.71 (0.95–3.09)	0.241 0.076
Physical abuse Total adversity Early adversity	1.07 (0.79–1.44) 0.71 (0.48–1.104)	0.664 0.076	0.95 (0.73–1.24) 0.81 (0.58–1.15)	0.693 0.208	**1.45** (1.03–2.04) 1.43 (0.92–2.23)	0.032 0.116
Sexual abuse Total adversity Early adversity	**1.89** (1.24–2.88) 1.69 (0.97–2.95)	0.003 0.063	**2.04** (1.41–2.96) 1.55(0.95–2.53)	<0.001 0.081	0.84 (0.54–1.32) 0.68 (0.36–1.30)	0.453 0.248
Bullying Total adversity Early adversity	0.95 (0.73–1.25) 1.12 (0.80–1.58)	0.729 0.515	1.12 (0.89–1.42) 1.28 (0.95–1.72)	0.324 0.110	0.99 (0.74–1.33) 1.03 (0.73–1.45)	0.967 0.862

Early adversity refers to exposure between birth and age 11; late adversity refers to exposure between ages 12 and 17; CI, confidence interval; aOR, adjusted odds ratio. Bold text indicates associations where *p* < 0.05.

a Adjusted for age, sex, ethnicity, years of education, country and other childhood adversities.

b Adjusted for age, sex, ethnicity, country and years of education.

Regarding different subtypes of childhood adversity, household discord, psychological abuse, sexual abuse and bullying were significantly associated with having an FEP. When considering early exposure to adversity, we obtain similar results with only early sexual abuse not being significantly associated with psychosis, whereas for later adversities, only sexual abuse and bullying remained strongly associated. All cannabis use measures were significantly related to psychotic disorder.

### Associations between childhood adversity and cannabis use

Associations between childhood adversity, total and early, and cannabis use are reported in Table 2 (panel B).

In our sample, household discord, both overall and early adversity, was significantly and positively associated with all measures of cannabis misuse. Early psychological abuse was significantly associated with lifetime cannabis use and cannabis potency, as well as total sexual abuse. Interestingly, exposure to bullying during adolescence was negatively associated with lifetime cannabis use (see online Supplementary Table S2).

### Mediating effects of cannabis use between childhood adversity and psychosis

The results of the mediation analyses (see Table 3) indicate that the relationship between household discord and psychosis is partially mediated by all measures of cannabis use, i.e. lifetime cannabis use (indirect effect coef. 0.078 s.e. 0.022, 17%), cannabis potency (indirect effect coef. 0.059 s.e. 0.018, 14%) and frequency of use (indirect effect coef. 0.117 s.e. 0.034, 42%). Lifetime cannabis use (indirect effect coef. 0.082 s.e. 0.045, 18%) and cannabis potency (indirect effect coeff. 0.078 s.e. 0.078, 19%) also mediated the relationship between sexual abuse and later psychosis.

**Table 3.** Mediation analyses displaying the total, direct, indirect effects and the percentage of total effect mediated between advertises and psychosis, via cannabis use patterns

Panel A. Main analyses on total exposure to adversity							
Childhood adversities and potential mediators	Total		Direct		Indirect		
	Coef. (s.e.)	*p* value	Coef. (s.e.)	*p* value	Coef. (s.e.)	% Mediated	*p* value
Household discord – total Lifetime cannabis use Cannabis potency Cannabis frequency	**0.451** (0.115) **0.411** (0.106) **0.404** (0.146)	<0.001 <0.001 0.006	**0.373** (0.116) **0.352** (0.106) 0.287 (0.146)	0.001 0.001 0.050	**0.078** (0.022) **0.059** (0.018) **0.117** (0.038)	17.25 14.37 28.94	<0.001 0.001 0.002
Sexual abuse – total Lifetime cannabis use Cannabis potency	0.460 (0.201) 0.418 (0.180)	0.022 0.020	0.377 (0.201) 0.338 (0.181)	0.061 0.061	**0.082** (0.034) **0.078** (0.029)	17.83 19.12	0.016 0.005
Panel B. Sensitivity analyses restricted to early exposure to adversity							

Childhood adversities and potential mediators	Total		Direct		Indirect		
	Coef. (s.e.)	*p* value	Coef. (s.e.)	*p* value	Coef. (s.e.)	% Mediated	*p* value
Household discord – early Lifetime cannabis use Cannabis potency Cannabis frequency	**0.581** (0.137) **0.518** (0.128) **0.530** (0.178)	<0.001 <0.001 0.003	**0.498** (0.138) **0.468** (0.128) **0.404** (0.178)	<0.001 <0.001 0.023	**0.083** (0.026) **0.050** (0.022) **0.126** (0.046)	14.26 9.63 23.77	0.002 0.024 0.006
Psychological abuse – early Lifetime cannabis use Cannabis potency	0.369 (0.256) 0.331 (0.229)	0.149 0.148	0.261 (0.256) 0.228 (0.230)	0.308 0.321	**0.108** (0.045) **0.104** (0.041)	29.16 31.28	0.017 0.011

Following Baron and Kenny criteria, mediation analyses have been conducted when the mediator is both associated with the predictor (adversities) and with the outcome simultaneously, based on analyses shown in Tables 1 and 2. Panel A refers to total exposure to adversity, while panel B shows the results of the sensitivity analyses restricted to early exposure to adversity (0–12 years).

Total effect, direct effect + indirect effect; direct effect, effect of the independent variable to the outcome variable; indirect effect, effect of the independent variable to the mediator and of the mediator on the outcome variable.

SE, standard error. Bold text indicates associations where *p* < 0.05.

**Figure 1.**
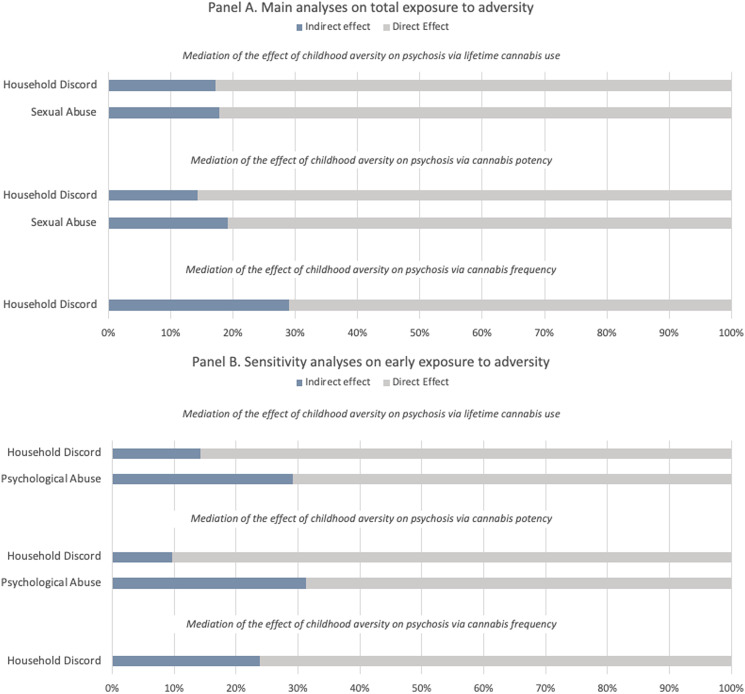
Proportion of the total effect of specific types of adversity on psychosis mediated via lifetime cannabis use, cannabis potency, and frequency of using cannabis. The blue portion of each bar indicates the percentage of the effect mediated (indirect effect). Panel A refers to the main analyses on total exposure to childhood adversity (0–17 years), Panel B refers to the sensitivity analyses restricted to early exposure to childhood adversity (0–12 years).

From the sensitivity analyses restricted to early (prior to age 12) exposure to adversity we obtained the following findings. Consistently, the association between early household discord and psychosis was mediated by lifetime cannabis use (indirect effect coef. 0.083 s.e. 0.026, 14%), cannabis potency (indirect effect coef. 0.050 s.e. 0.022, 10%) and cannabis frequency (indirect effect coef. 0.126 s.e. 0.46, 24%). Both lifetime cannabis use (indirect effect coeff. 0.108 s.e. 0.045, 29%) and cannabis potency (indirect effect coeff. 0.104 s.e. 0.041, 31%) mediated the effect of early psychological abuse. The different mediation models are illustrated in Fig. 1. Mediation analyses on late exposure to adversity (12–17 years) are reported in the online Supplementary Materials (see Table S3 and Fig. S5). Although not reaching the statistical significance threshold, it is still worth noticing the negative effect that lifetime cannabis use seems to have on the association between late bullying and psychosis (indirect effect coeff. −0.082 s.e. 0.044, −14%).

## Discussion

Using data from a large multicentre case–control study of first-episode psychosis, we examined whether cannabis use, in different forms, mediated the relationship between childhood adversity subtypes and the risk of developing a psychotic disorder.

Our findings suggest that around a fifth of the association between household discord before 18 and psychosis (% of total effect mediated ranging from 14% to 29%) was mediated by cannabis use, high potency cannabis and frequency of use, with slightly smaller mediating effects found when household discord was considered as occurring before age of 12 (% of total effect mediated ranging from 10% to 24%). The risk of developing psychosis after being exposed to psychological abuse was mediated by lifetime cannabis use and cannabis potency, when the adversity occurred early (around 30% of total effect mediated). Lifetime cannabis use and cannabis potency also mediated around 18% of the link between sexual abuse and psychotic disorder. Surprisingly, lifetime cannabis use had a slightly negative mediating effect on the relationship between late bullying and psychosis. This is due to a negative link between those individuals that have faced such adversity and risk of using cannabis. No mediation models could be tested for physical abuse, because interestingly no association between such type of trauma and psychosis was found in our sample. This might depend on a too broad definition utilised which did not allow us to discriminate whether exposure to physical abuse might lead to an increased risk of developing psychosis.

### Strengths and limitations

The relationship between childhood adverse experiences and later substance misuse has received growing attention in the last few years, providing evidence of increased drug use in patients who were exposed to abuse in childhood (Schäfer & Fisher, 2011). Some recent studies focused on the interaction between childhood adversity, cannabis use and psychosis, reporting a significantly greater risk for psychotic outcomes (Houston et al., 2008; Sideli et al., 2018). However, the novelty and major strength of the current study is the use of mediation analysis to elucidate the mechanisms underlying the nature of the association. To demonstrate mediation, one must establish strong relationships between the predictor, the mediator and a criterion variable, and previous studies failed to do so (Bebbington et al., 2011).

Furthermore, the specific questionnaire we used allows a detailed assessment of lifetime patterns of cannabis use, including age at first use, frequency and duration of use, and the specific type of cannabis used. Although a clear dose–response association has consistently been shown between cannabis use and the risk of developing psychosis, only few studies have collected detailed data on the pattern of cannabis use or its potency (Di Forti et al., 2019). Similarly, the measure utilised to assess childhood adversity allows the characterisation of different types of adversity stratified by age of exposure.

Moreover, the sample utilised in the present study was a well-characterised sample of recent-onset patients presenting for the first time with psychosis and representative controls. Among cannabis users, we selected participants who started using cannabis after age 12 in order to decrease the risk of reverse causation between cannabis use and exposure to adversity.

Having acknowledged these strengths, it is also important to recognise the limitations of our work. Most importantly, although we tried to diminish the risk of reverse causation between exposure to adversity cannabis use by looking at childhood and adolescence separately and excluding cannabis used prior to age 12, we cannot exclude the possibility that some exposures (i.e. late adolescent bullying or abuse) occurred slightly after or at the same than cannabis initiation. Case–control data are also collected retrospectively, and like all such data, may be subject to some level of recall bias, especially in the collection of traumatic experiences in people with psychosis (Howard, 1993). Given the inherent limitations of using case–control data to investigate mediation, we did not attempt to implement more sophisticated mediation models, such as causal mediation models in the present paper. Prospective, longitudinal studies are thus required to disentangle the directions of these associations using stronger causal inference methods. Additionally, we were unable to undertake bootstrapping, limiting our understanding of the accuracy of our inference. Another limitation regards the range of confounders that have been taken into account. We could not account for cognitive function which in multiple studies has shown to be associated with childhood adversity exposure (Vargas et al., 2019). Although we adjusted by the most relevant confounders in the field of trauma, cannabis and psychosis literature, the interplay between trauma and other environmental variables such as migration or discrimination is difficult to disentangle and was not the focus of the current paper. A recent publication on this topic from our group is already available (D'Andrea et al., 2022). We hope other factors such as the genetic influence in the form of family history and polygenic risk scores can be explored in the future and has not been focus of the current work either.

Finally, the number of statistical tests carried out was significantly essential; thus, we cannot confidently rule out the possibility that some of the associations might have been due to type I errors.

### Specific role of cannabis consumption in those exposed to household discord

The mediational role of cannabis use was particularly robust for experiences of household discord, especially when the exposure occurred in childhood, relative to other types of adversity, such as psychological, physical and sexual abuse. However, the risk of developing psychosis tends to be higher in those exposed to more severe experiences of abuse (Varese et al., 2012). Thus, this suggests that the association with psychosis in those exposed to such experiences might be mediated by other variables that we did not explore in this study, such as low mood, PTSD-related symptoms, negative schemas about the self, the world and others (Alameda et al., 2020).

### Cannabis use during adolescence, an important insult in a critical period of vulnerability

Growing evidence has pointed out adolescence as a particularly vulnerable developmental period during which exposure to cannabis might lead to deleterious consequences, such as developing psychosis (Arseneault, Cannon, Witton, & Murray, 2004; Hall, 2006). The maturational processes occurring during puberty and adolescence are necessary for adult behaviour. Thus, it is not surprising that immature individuals seem to be particularly susceptible to the exposure of cannabis. Cannabis exposure during pubertal development can lead to abnormal social behaviour and anhedonia in adulthood (Skumlien et al., 2021), which are also symptoms of psychosis. Although the association between early cannabis use and subsequent problems may be due, in part, to common risk factors, monitoring the age of initial cannabis use remains important.

### Future perspective and clinical implications

These results have important therapeutic implications suggesting that cannabis use may be a useful preventive intervention target in children exposed to household discord, particularly in those exposed prior to the age of 12. At a clinical level, our results support the need for a comprehensive assessment of childhood adversity and cannabis use history in first-episode psychosis patient. Young people with a history of household discord could potentially be targeted for psychotherapeutic or psycho-educational interventions regarding the risks of cannabis use, particularly during adolescence. Current findings outline the need for future research on the role of cannabis use in the association between childhood adversity and psychosis. The results should be replicated using a prospective design to clarify the temporal relationship between risk factors and psychosis, excluding the effect of reverse causality and recall bias.

In addition, research analysing the interaction between environmental risk factors (including migration, urbanicity, substance misuse and recent life events) and genetics is needed to better investigate the aetiology of psychosis. For instance, direct measures of genetic variation and family history may help in evaluating adversity and cannabis-related risk of psychosis in the context of genetic susceptibility. This has potential implications for prevention, for example, in predicting those at risk of psychosis who can ultimately benefit from specific clinical interventions.

## Supporting information

Trotta et al. supplementary material 1 Trotta et al. supplementary material

Trotta et al. supplementary material 2 Trotta et al. supplementary material

## Data Availability

The data that support the findings of this study is available on request from the corresponding author, G. T.

## Supplementary material

The supplementary material for this article can be found at https://doi.org/10.1017/S0033291723000995.
