# Supplementary material for: Cannabis use as a potential mediator between childhood adversity and first-episode psychosis: results from the EU-GEI case–control study
Source: Psychol Med. 2023 May 4;53(15):7375–84. doi: 10.1017/S0033291723000995 (PMC10719680; doi:10.1017/S0033291723000995)
Supplement: Trotta et al. supplementary material 1 — Trotta et al. supplementary material [file S0033291723000995sup001.docx]

**SUPPLEMENTARY MATERIALS TO ACCOMPANY:**

Cannabis use as a potential mediator between childhood adversity and first-episode psychosis: Results from the EU-GEI case-control study

[1. Supplementary Methods 3](#_Toc127547141)

[*1.1* *Measures of cannabis use* 3](#_Toc127547142)

[*1.2* *Mediation model* 4](#_Toc127547143)

[2. Supplementary Results 5](#_Toc127547144)

[*2.1* *Recruitment flow charts* 5](#_Toc127547145)

[*2.2* *Distribution of age at first using cannabis by case-control status* 6](#_Toc127547146)

[*2.3* *Logistic Regressions Between Late Childhood Adversities and Psychosis* 7](#_Toc127547147)

[*2.4* *Logistic Regressions Between Late Childhood Adversities and Cannabis Use* 7](#_Toc127547148)

[*2.5* *Mediation Analyses restricted to Late exposure to Adversity* 8](#_Toc127547149)

[3. Supplementary discussion 9](#_Toc127547150)

[*3.1* *The negative link with cannabis consumption in those exposed to bullying* 9](#_Toc127547151)

[4. Supplementary References 10](#_Toc127547152)

The supplementary material has been provided by the authors to give readers additional information about their work.

# Supplementary Methods

## *Measures of cannabis use*

Detailed history of cannabis use and other recreational drugs were collected using an updated version of the Cannabis Experiences Questionnaire, further revised for the EU-GEI study (CEQ_EU-GEI_).(di Forti et al., 2019) For the purpose of this study, we included three variables in our analyses (1) lifetime cannabis use, which refers to use of cannabis at any time in the lifespan, (2) cannabis potency, and (3) frequency of use. While the variable age at first using cannabis was used to exclude those who started smoking cannabis before adolescence (12 years).

The potency variable was created, consistently with previous papers in the field, using a cut-off of 10% THC concentration. The participants were asked to name in their own language the type of cannabis mostly used, and their responses were compared with the EMCDDA and the National data on cannabis potency quoted to estimate THC levels.(‘ESPAD Report 2019 — Results from the European School Survey Project on Alcohol and Other Drugs | Www.Emcdda.Europa.Eu’, n.d.) The low-potency cannabis category (THC < 10%) included hash/resin from UK and Italy, imported herbal cannabis from UK, Italy, Spain and France, Brazilian marijuana and hash and the Dutch *Geimporteerde Wiet*. The high-potency category (THC ≥ 10%) included all the other types reported by the study participants in their original language street names such as: UK home-grown skunk/*sensimilla*, UK Super Skunk, Italian home-grown skunk/sensimilla, Italian Super Skunk, the Dutch *Nederwiet*, *Nederhasj* and *geimporteerde hasj*, the Spanish and French Hashish (from Morocco), Spanish home-grown *sensimilla*, French home-grown skunk/*sensimilla*/super-skunk and Brazilian skunk.

The variable frequency of use was generated on the question “Describe how often from the following options” and originally consisted in seven different categories: a) I used it only once or twice; b) about once a year; c) few times a year; d) about one/twice a month; e) about once a week; f) more than once a week; g) every day. In this study we grouped the answers in three categories: a) used never or occasionally (less than once a week); b) used more than once a week (but less than daily); c) used daily.

## *Mediation model*

**
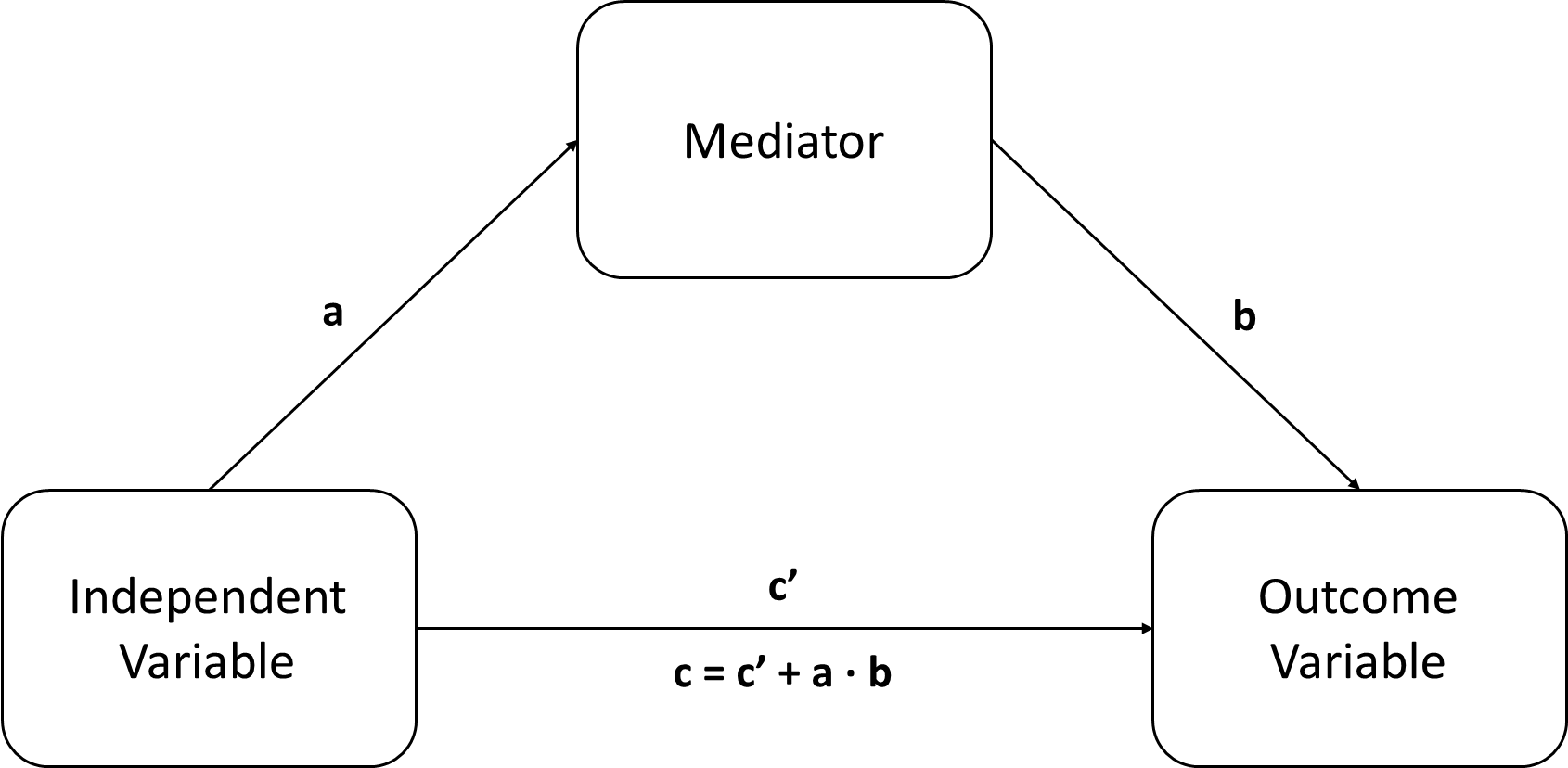
**

**Fig S1.** *Mediation model.* Path a = effect of the independent variable to the mediator; path b = effect of the mediator to the outcome variable; a ∙ b = indirect effect of the mediator to the outcome variable; path c’= direct effect of the independent variable to the outcome variable; path c = total effect (direct effect + indirect effect).

In our sample mediation analysis was performed in order to study whether cannabis use (mediator) mediated the relationship between trauma, i.e., household discord, psychological, physical and sexual abuse (independent variables), and psychosis (outcome variable).

# Supplementary Results

## Recruitment flow charts

**
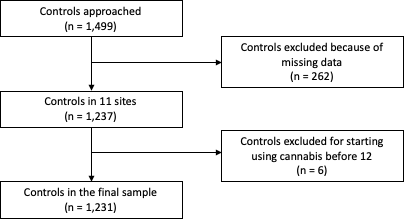
**

**Fig S2.** *Recruitment flow chart for controls.*

**
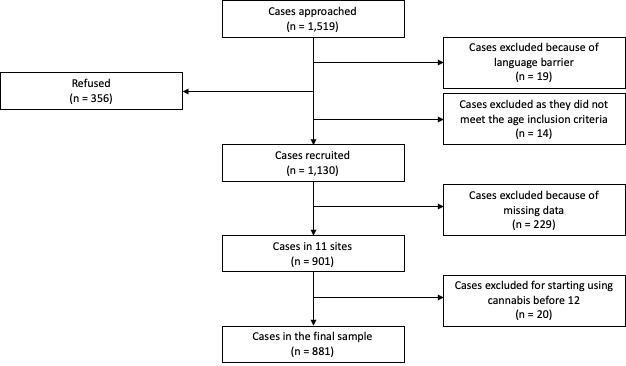
**

**Fig S3.** *Recruitment flow chart for cases.*

## Distribution of age at first using cannabis by case-control status


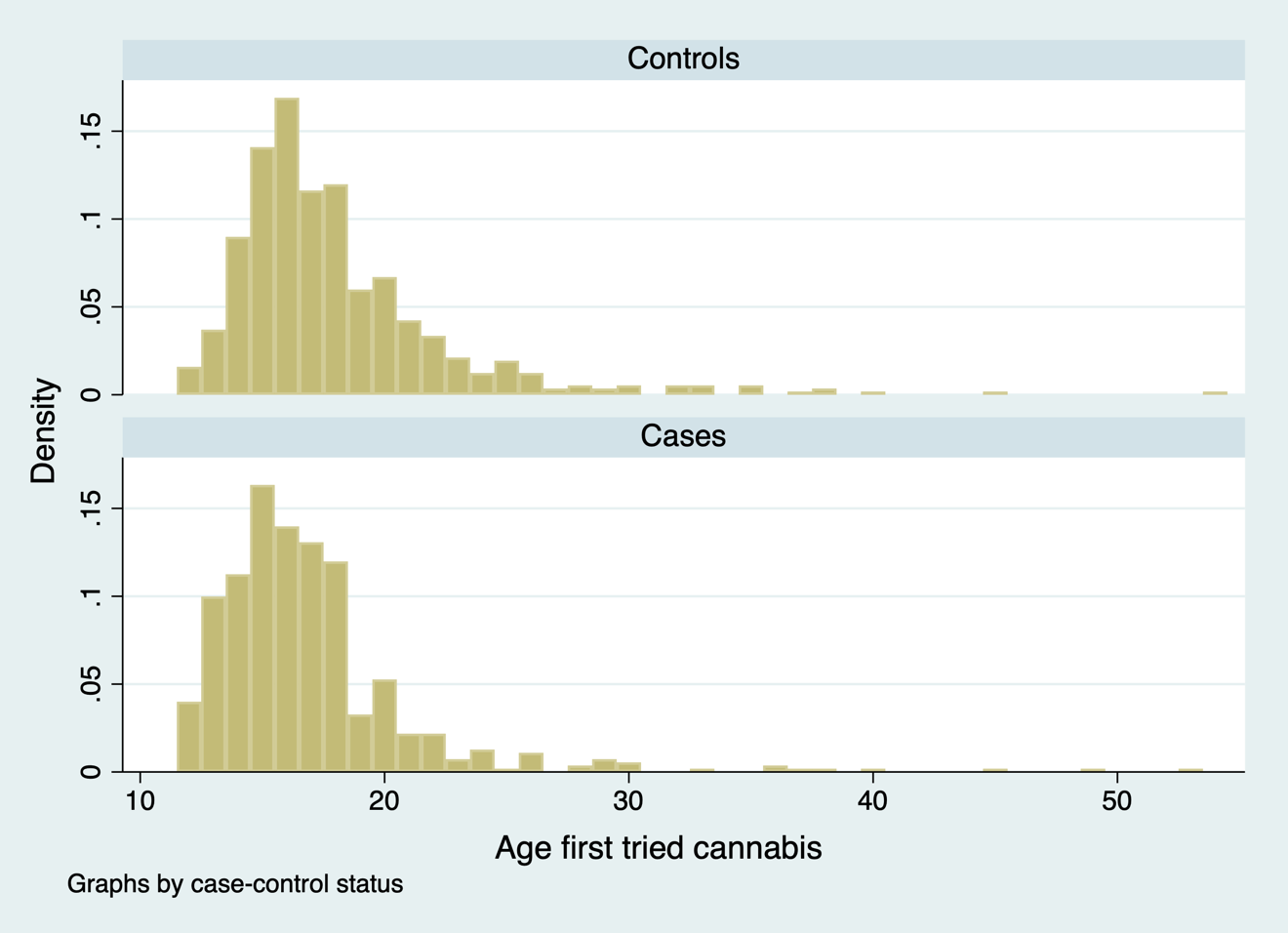


**Fig S4.** *Histrograms showing age at first using cannabis by case-control status.*

## Logistic Regressions Between Late Childhood Adversities and Psychosis

**Table S1.** *Associations Between Late Childhood Adversities and Psychotic Disorder*

|  | aOR (95% CI) | *p-*value |
| --- | --- | --- |
| Household discord  Late adversity | 1.29 (0.81-2.05) | 0.279 |
| Psychological abuse  Late adversity | 1.64 (0.54-4.97) | 0.385 |
| Physical abuse  Late adversity | 0.78 (0.38-1.60) | 0.500 |
| Sexual abuse  Late adversity | **3.34** (1.01-11.09) | 0.049 |
| Bullying  Late adversity | **1.66** (1.05-2.61) | 0.029 |

Late adversity refers to exposure between ages 12 and 17; CI = confidence interval; aOR = odds ratio adjusted for age, sex, ethnicity, years of education, country and other childhood adversities. Bold text indicates associations where p<0.05.

## Logistic Regressions Between Late Childhood Adversities and Cannabis Use

**Table S2.** *Associations between Late Childhood Adversities and Cannabis Use*

|  | LIFETIME CANNABIS USE | | CANNABIS POTENCY | | CANNABIS FREQUENCY | |
| --- | --- | --- | --- | --- | --- | --- |
|  | **aOR (95% CI)** | ***p-*value** | **aOR (95% CI)** | ***p-*value** | **aOR (95% CI)** | ***p-*value** |
| Household discord  Late adversity | 1.69 (0.96-2.96) | 0.069 | **2.12** (1.31-3.41) | 0.002 | 1.47 (0.79-2.75) | 0.228 |
| Psychological abuse  Late adversity | 1.53 (0.42-5.55) | 0.516 | 1.19 (0.38-3.82) | 0.759 | 1.19 (0.23-6.19) | 0.832 |
| Physical abuse  Late adversity | **2.40** (1.05-5.51) | 0.039 | 1.31 (0.60-2.83) | 0.498 | 1.00 (0.36-2.79) | 0.995 |
| Sexual abuse  Late adversity | **4.39** (1.07-18.04) | 0.040 | 2.04 (0.57-7.24) | 0.272 | 6.15 (1.17-32.28) | 0.032 |
| Bullying  Late adversity | **0.54** (0.30-0.96) | 0.035 | 0.78 (0.49-1.26) | 0.309 | 0.64 (0.32-1.26) | 0.198 |

CI = confidence interval; aOR = Odds ratio adjusted for age, sex, ethnicity, years of education, country and other childhood adversities. Bold text indicates associations where p<0.05.

## Mediation Analyses restricted to Late exposure to Adversity

**Table S3.** *Mediation analyses displaying the total, direct, indirect effects and the percentage of total effect mediated between advertises and psychosis, via cannabis use patterns.*

| Childhood Adversities  and Potential Mediators | TOTAL | | DIRECT | | INDIRECT | | |
| --- | --- | --- | --- | --- | --- | --- | --- |
|  | **Coef. (SE)** | ***p-*value** | **Coef. (SE)** | ***p-*value** | **Coef. (SE)** | **% Mediated** | ***p-*value** |
| Sexual abuse - Late  Lifetime cannabis use  Cannabis frequency | **1.548** (0.665)  0.897 (0.828) | 0.020  0.278 | **1.388** (0.667)  0.566 (0.831) | 0.037  0.496 | 0.159 (0.087)  0.331 (0.177) | 10.31  36.94 | 0.072  0.062 |
| Bullying - Late  Lifetime cannabis use | **0.605** (0.265) | 0.023 | **0.687** (0.267) | 0.010 | -0.082 (0.044) | -13.56 | 0.061 |

Sensitivity analyses were restricted to late exposure to adversity (12-17 years). Following Baron and Kenny criteria, mediation analyses have been conducted when the mediator is both associated with the predictor (late adversities) and with the outcome simultaneously, based on analyses shown in Tables S1, and S2.

Total effect = direct effect + indirect effect; Direct effect = effect of the independent variable to the outcome variable; Indirect effect = effect of the independent variable to the mediator and of the mediator on the outcome variable.

SE = standard error. Bold text indicates associations where p<0.05.

**
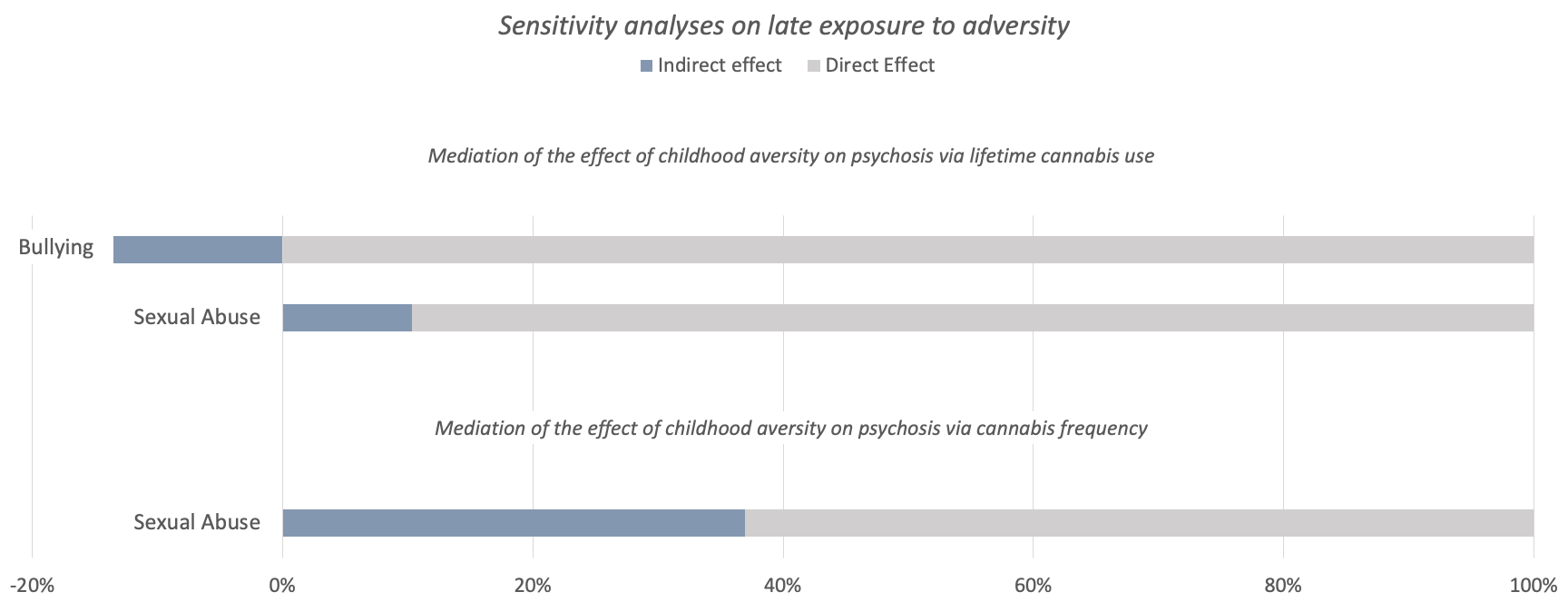
**

**Fig S5.** *Proportion of the total effect of specific types of adversity on psychosis mediated via lifetime cannabis use, and frequency of using cannabis.* The blue portion of each bar indicates the percentage of the effect mediated (indirect effect). The graph refers to the sensitivity analyses restricted to late exposure to childhood adversity (12-17 years).

**Table S4.** *Mediation analyses displaying the total, direct, indirect effects and the percentage of total effect mediated between advertises and psychosis, via cannabis use patterns.*

| *Panel A. Main analyses on total exposure to adversity* | | | | | | | |
| --- | --- | --- | --- | --- | --- | --- | --- |
| Childhood Adversities  and Potential Mediators | **TOTAL** | | **DIRECT** | | **INDIRECT** | | |
|  | **aOR (CI)** | ***p-*value** | **aOR (CI)** | ***p-*value** | **aOR (CI)** | **% Mediated** | ***p-*value** |
| Household discord - Total  Lifetime cannabis use  Cannabis potency  Cannabis frequency | **1.57** (1.25 – 1.97)  **1.51** (1.23 – 1.86)  **1.50** (1.13 – 1.99) | <0.001  <0.001  0.006 | **1.45** (1.16 – 1.82)  **1.42** (1.16 – 1.75)  1.33 (0.99 – 1.77) | 0.001  0.001  0.050 | **1.08** (1.04 – 1.13)  **1.06** (1.02 – 1.10)  **1.12** (1.04 – 1.21) | 17.25  14.37  28.94 | <0.001  0.001  0.002 |
| Sexual abuse - Total  Lifetime cannabis use  Cannabis potency | **1.58** (1.07 – 2.35)  **1.52** (1.07 – 2.16) | 0.022  0.020 | 1.46 (0.98 – 2.16)  1.40 (0.98 – 1.99) | 0.061  0.061 | **1.09** (1.02 – 1.16)  **1.08** (1.02 – 1.15) | 17.83  19.12 | 0.016  0.005 |
| *Panel B. Sensitivity analyses restricted to early exposure to adversity* | | | | | | | |
| Childhood Adversities  and Potential Mediators | **TOTAL** | | **DIRECT** | | **INDIRECT** | | |
|  | **aOR (CI)** | ***p-*value** | **aOR (CI)** | ***p-*value** | **aOR (CI)** | **% Mediated** | ***p-*value** |
| Household discord - Early  Lifetime cannabis use  Cannabis potency  Cannabis frequency | **1.79** (1.37 – 2.34)  **1.68** (1.31 – 2.16)  **1.70** (1.20 – 2.41) | <0.001  <0.001  0.003 | **1.65** (1.26 – 2.16)  **1.60** (1.24 – 2.05)  **1.50** (1.06 – 2.12) | <0.001  <0.001  0.023 | **1.09** (1.03 – 1.14)  **1.05** (1.01 – 1.10)  **1.13** (1.04 – 1.24) | 14.26  9.63  23.77 | 0.002  0.024  0.006 |
| Psychological abuse – Early  Lifetime cannabis use  Cannabis potency | 1.45 (0.88 – 2.39)  1.39 (0.89 – 2.18) | 0.149  0.148 | 1.30 (0.79 – 2.15)  1.26 (0.80 – 1.97) | 0.308  0.321 | **1.11** (1.02 – 1.22)  **1.11** (1.02 – 1.20) | 29.16  31.28 | 0.017  0.011 |
| *Panel C. Sensitivity analyses restricted to late exposure to adversity* | | | | | | | |
| Childhood Adversities  and Potential Mediators | **TOTAL** | | **DIRECT** | | **INDIRECT** | | |
|  | **aOR (CI)** | ***p-*value** | **aOR (CI)** | ***p-*value** | **aOR (CI)** | **% Mediated** | ***p-*value** |
| Sexual abuse - Late  Lifetime cannabis use  Cannabis frequency | **4.70** (1.28 – 17.31)  2.45 (0.48 – 12.43) | 0.020  0.278 | **4.01** (1.09 – 14.80)  1.76 (0.35 – 8.98) | 0.037  0.496 | 1.17 (0.99 – 1.40)  1.39 (0.98 – 1.97) | 10.31  36.94 | 0.072  0.062 |
| Bullying - Late  Lifetime cannabis use | **1.83** (1.088 – 3.08) | 0.023 | **1.99** (1.18 – 3.36) | 0.010 | 0.92 (0.85 – 1.00) | -13.56 | 0.061 |

Panel A refers to total exposure to adversity, while Panel B and C shows the results of the sensitivity analyses restricted to early exposure to adversity (0-12 years) and later exposure to adversity (12-18 years), respectively. Total effect = direct effect + indirect effect; Direct effect = effect of the independent variable to the outcome variable; Indirect effect = effect of the independent variable to the mediator and of the mediator on the outcome variable.

aOR = odd ratio adjusted for age, sex, ethnicity, years of education and country. CI = confidence interval. Bold text indicates associations where p<0.05.

#

# Supplementary discussion

## The negative link with cannabis consumption in those exposed to bullying

Interestingly, the relationship between bullying occurring in adolescence and cannabis exposure appeared to be negative. Indeed, adolescents exposed to this type of adversity may be at higher risk of developing a psychotic disorder despite being less prone to smoke cannabis (Singham et al., 2017). This could be explained by the possibility that bullied adolescents remain isolated and withdrawn following such adversity, and thus are not able to easily access this substance. This subjects could be more prone to develop psychosis independently of cannabis and its harmful biological effect (Mayet et al., 2010; Velikonja et al., 2015). For example, the underlying genetic vulnerability could play a role in these different ways to cope with bullying. However, these are speculations and should be further explored, as well as other potential mediating factors that may operate in this group (e.g., social defeat, loneliness and social support) (Alameda et al., 2019).

# Supplementary References

Alameda, L., Rodriguez, V., Ewan, C., Aas, M., Trotta, G., Spinazzola, E., … M. Murray, R. (2019). A Systematic Review on Psychological and Biological Mediators Between Adversity and Psychosis: Potential Targets for Treatment. *MedRxiv*, 2019.12.14.19014506. https://doi.org/10.1101/2019.12.14.19014506

di Forti, M., Quattrone, D., Freeman, T. P., Tripoli, G., Gayer-Anderson, C., Quigley, H., … van der Ven, E. (2019). The contribution of cannabis use to variation in the incidence of psychotic disorder across Europe (EU-GEI): a multicentre case-control study. *The Lancet. Psychiatry*, *6*(5), 427–436. https://doi.org/10.1016/S2215-0366(19)30048-3

ESPAD Report 2019 — Results from the European School Survey Project on Alcohol and Other Drugs | www.emcdda.europa.eu. (n.d.). Retrieved 23 November 2021, from https://www.emcdda.europa.eu/publications/joint-publications/espad-report-2019_en

Mayet, A., Legleye, S., Chau, N., & Falissard, B. (2010). The mediation role of licit drugs in the influence of socializing on cannabis use among adolescents: A quantitative approach. *Addictive Behaviors*, *35*(10), 890–895. https://doi.org/10.1016/j.addbeh.2010.06.001

Singham, T., Viding, E., Schoeler, T., Arseneault, L., Ronald, A., Cecil, C. M., … Pingault, J. B. (2017). Concurrent and Longitudinal Contribution of Exposure to Bullying in Childhood to Mental Health: The Role of Vulnerability and Resilience. *JAMA Psychiatry*, *74*(11), 1112–1119. https://doi.org/10.1001/jamapsychiatry.2017.2678

Velikonja, T., Fisher, H. L., Mason, O., & Johnson, S. (2015). Childhood trauma and schizotypy: A systematic literature review. *Psychological Medicine*, *45*(5), 947–963. https://doi.org/10.1017/S0033291714002086
